# The translational paradox of AI in hepatocellular carcinoma: from algorithmic over-engineering to real-world clinical utility

**DOI:** 10.3389/fonc.2026.1848190

**Published:** 2026-05-20

**Authors:** Chen Li, Yuka Yanase, Ming-Quan Pang

**Affiliations:** 1 Qinghai Research Key Laboratory for Echinococcosis, Department of Hepatopancreatobiliary Surgery, Affiliated Hospital of Qinghai University, Xining, China; 2 Department of Breast Surgery, Graduate School of Medicine, Teikyo University, Tokyo, Japan

**Keywords:** artificial intelligence, hepatocellular carcinoma, spatial transcriptomics, unsupervised domain adaptation, vision foundation models

## Abstract

Synthesizing recent literature from 2025 to 2026, this mini-review critically evaluates the methodological evolution of artificial intelligence (AI) in hepatocellular carcinoma (HCC) from static pattern recognition to mechanism-driven spatial and imaging diagnostics. We expose a critical translational paradox: although self-supervised Vision Foundation Models (VFMs), unsupervised domain adaptation for H&E-based virtual molecular profiling, and AI-synergized spatial transcriptomics theoretically decode intratumoral heterogeneity and mitigate cross-etiology domain shifts, these complex architectures remain unvalidated in real-world multi-scanner cohorts. Conversely, robust comparative analyses challenge the prevailing multi-modal narrative by demonstrating that algorithmic utility is highly data-dependent: although complex AI is crucial for high-dimensional settings, traditional Cox models remain highly robust and competitive compared to highly engineered large language models (LLMs) in low-dimensional survival prediction, indicating that algorithmic complexity does not inevitably equal clinical utility. To break this translational impasse, this mini-review argues that the field must urgently pivot away from fragile *post-hoc* explainability toward inherently interpretable architectures—such as concept bottleneck models (CBMs)—and calls for the rigorous integration of these transparent systems into Phase II “window-of-opportunity” randomized trials and dynamic regulatory evaluation frameworks within the next 24 months.

## 1 Introduction

**Figure 1:**
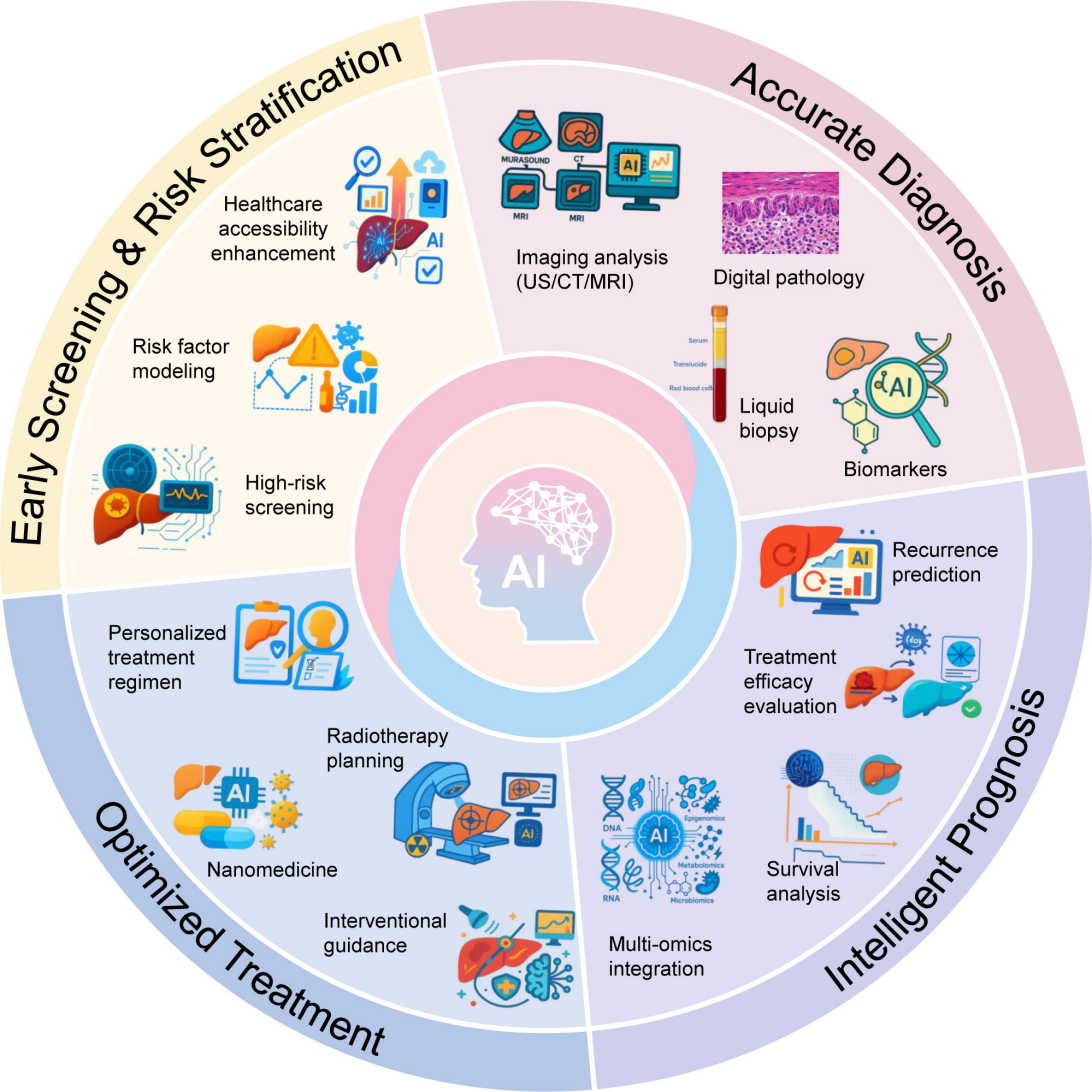
Application of artificial intelligence in multiple related fields of hepatocellular carcinoma.

HCC is the most common primary liver malignancy and a leading global cause of cancer-related mortality (1). Currently, the clinical management of HCC relies heavily on standardized staging systems and morphological assessments. However, these conventional paradigms are fundamentally hindered by interobserver variability and an inability to noninvasively capture intratumoral heterogeneity (ITH). Moreover, their rigid decision algorithms frequently fail to accommodate individualized tumor biology and emerging predictive biomarkers (2). The rapid integration of AI presents a critical opportunity to refine HCC screening protocols and circumvent these diagnostic bottlenecks (3, 4). AI is now widely used in many areas of HCC (**Figure 1**).

Between early 2025 and 2026, the landscape of computational oncology underwent a pivotal methodological evolution. Transitioning from mere proof-of-concept models to clinically actionable instruments, AI now facilitates deep, multidimensional data extraction across both macroscopic and microscopic scales. This mini-review critically evaluates the vanguard applications of AI in HCC, focusing on the most translationally relevant domains: the fundamental algorithmic evolution for resolving domain shift in medical imaging AI, digital pathology-driven virtual molecular profiling, the pragmatic recalibration of advanced survival prediction models, and the synergistic technical pathways integrating spatial transcriptomics with AI to decode tumor heterogeneity.

A comprehensive review of the literature was performed utilizing MEDLINE/PubMed and Web of Science databases with the end of search date being the March 1, 2026. The MeSH terms “Artificial Intelligence,” “Liver Cancer,” “Hepatocellular Carcinoma,” “Machine Learning,” and “Deep Learning” were searched in the title and/or abstract.

## 2 Limitations of AI in HCC medical imaging and structural vulnerabilities to domain shift

Contrast-enhanced computed tomography (CT) and magnetic resonance imaging (MRI) remain the cornerstones of HCC surveillance. Although AI models excel in automated tumor delineation—such as the SALSA framework, which recently surpassed inter-expert consensus in liver tumor segmentation (5)—they exhibit profound structural vulnerabilities when integrated into real-world clinical workflows.

Specifically, Zhang et al. (6) reported on Liver Imaging Reporting and Data System (LI-RADS) classification assisted by large language models (LLMs), such as DeepSeek. It is crucial to delineate that, in this context, the LLM executes a natural language processing (NLP) task on unstructured radiological text reports, rather than a pixel-based computer vision (CV) task. Despite demonstrating high theoretical accuracy, this model proved extremely sensitive to minor variations in reporting lexicons across different medical institutions. This highlights an inherent limitation of medical AI: when generic LLMs process real-world clinical texts—which lack universally standardized ontologies and are heavily context-dependent—they are highly susceptible to “semantic drift,” inevitably leading to misclassification.

Furthermore, within the realm of pixel-based CV, although numerous imaging AI models have demonstrated high internal validity for diverse diagnostic tasks (**Table 1**), “domain shift” remains a primary barrier to clinical translation; their performance frequently degrades when exposed to external datasets featuring differing scanner protocols or contrast agent dynamics. Recent studies have extensively utilized gadobenate dimeglumine (Gd-BOPTA) MRI-based multilayer perceptron (MLP) deep learning models (7) or radiopathomics fusion models (8) to noninvasively predict vessels encapsulating tumor clusters (VETC). A related meta-analysis confirmed their excellent internal validity (area under the receiver operating characteristic curve [AUC] > 0.85) (9). Additionally, contrast-enhanced CT-based radiomic habitat analysis can concurrently predict programmed cell death ligand 1 (PD-L1) expression and early recurrence (10–12). However, despite parallel advances in tasks such as distinguishing intrahepatic cholangiocarcinoma (13), these algorithms inevitably overfit to the specific imaging parameters of their originating institutions.

**Table 1:** AI applications in HCC medical imaging.

Reference	Year	Algorithms	Sample size	Aim	Best results
Balaguer-Montero et al. (5)	2025	DL	n=1,306	Automate liver tumor delineation tool	Model vs. Expert: (DSC 0.760 vs.0.800)
Zhang et al. (6)	2025	LLM	n=426	DSV3 vs.Radiologists: LI-RADS	ACC = 0.80(LR-4) ACC = 0.86(LR-5)
Gu et al. (7)	2025	DL	n=230	Gd-BOPTA-MLP predicts HCC VETC	AUC = 0.912 Se = 0.917 Sp = 0.850
Yu et al. (8)	2025	ML/DL	n=399	Radiopathomics predicts VETC, survival	AUC = 0.88 C-index = 0.77
Shui et al. (9)	2026	ML/DL	n= 6,755	Evaluate ML-based VETC detection	AUC = 0.83(ML) AUC = 0.81(DL)
Wang et al. (10)	2025	ML/DL	n=162	Noninvasively predict PD-L1, VETC	AUC = 0.705
Zhang et al. (11)	2025	ML	n=344	Preoperative early recurrence prediction	AUC = 0.896
Chen et al. (12)	2025	ML/DL	n=539	Assess CK19 and survival noninvasively	AUC = 0.863
Qian et al. (13)	2025	ML	n=189	ML-Radiomics differentiates CCA	AUC = 0.931

DL, Deep Learning; LLM, Large Language Model; ML, Machine Learning; DSC, Dice Similarity Coefficient; DSV3, Deep Supervised Version 3; LI-RADS, Liver Imaging Reporting and Data System; LR-4, LI-RADS 4 (Probably Hepatocellular Carcinoma); LR-5, LI-RADS 5 (Definitely Hepatocellular Carcinoma); Gd-BOPTA-MLP, Gadobenate dimeglumine; HCC, Hepatocellular carcinoma; VETC, Vessels Encapsulating Tumor Clusters; PD-L1, Programmed Death-Ligand 1; CK19, Cytokeratin 19; CCA, Cholangiocarcinoma; ACC, Accuracy; AUC, Area Under the Curve; C- index, Concordance Index.

Contemporary medical imaging AI predominantly relies on retrospective, small-sample, single-institution radiomic features. Traditional solutions, such as establishing multicenter databases, fail to resolve the fundamental generalization crisis caused by data heterogeneity at the algorithmic root. The current breakthrough lies in reducing reliance on costly, manually annotated supervised learning by comprehensively exploring self-supervised learning (SSL) and vision foundation models (VFMs, e.g., the medical adaptation of Segment Anything). By pretraining on massive, heterogeneous, unannotated medical images, these cutting-edge models learn the universal manifold structures of liver anatomy and tumor growth. Consequently, when encountering novel scanner protocols, VFMs theoretically require only few-shot learning to resist domain shift. This represents a critical infrastructural evolution for the clinical deployment of imaging AI.

## 3 Digital pathology and H&E-based virtual molecular profiling

Traditional histopathological review of hematoxylin and eosin (H&E)–stained slides is inherently qualitative. Recently, AI has accelerated the transition toward quantitative computational pathology, enabling “virtual molecular profiling” directly from standard slides (14). This advancement is particularly relevant for predicting HCC development driven by emerging etiologies, such as metabolic dysfunction-associated steatotic liver disease (MASLD) (15).

Regarding AI-driven identification of tumor polyploidy, Matsuura et al. developed a deep learning image recognition model capable of selectively identifying highly aggressive polyploid HCC directly from standard H&E images, offering an algorithmic alternative to expensive fluorescence *in situ* hybridization (FISH) testing (16). Simultaneously, deep learning frameworks can now infer outcome-related molecular profiles and predict microvascular invasion (mVI) based solely on the spatial topological arrangement of stromal components and lymphocytes on H&E slides (17). Moreover, integrated digital pathology models further merge these pathomic features with baseline clinical parameters, significantly refining the precision of recurrence predictions (18) (**Table 2**).

**Table 2:** AI in digital pathology and virtual molecular profiling in HCC.

Reference	Year	Algorithms	Sample size	Aim	Best results
Zhang et al. (14)	2025	ML/DL	3.1M WSIs	Evaluate AI in digital pathology	AUC = 0.95 ACC = 0.96
Nakatsuka et al. (15)	2025	ML/CNN	n= 1,328	Predict HCC risk in SLD	AUC = 0.84
Matsuura et al. (16)	2025	DL	n=399	HCC polyploidy diagnosis, prognosis	AUC = 0.887
Seraphin et al. (17)	2025	DL	n=781	H&E predicts molecular, mVI	AUC = 0.81
Wang et al. (18)	2026	ML	n= 294	Pathomics predicts HCC recurrence	AUC = 0.893 NRI = 0.630 IDI = 0.419
Zhuo et al. (19)	2026	ML	n=1050	Link OTA to HCC mechanisms	AUC = 0.963
Gao et al. (20)	2025	ML	n=642	Investigate AFB1-induced HCC mechanisms	AUC = 0.896
Han et al. (21)	2025	ML/DL	TCGA/GEO	AI-driven ferroptosis research in HCC	Study proved ferroptosis-guided AI optimizes HCC prognosis.

ML, Machine Learning; DL, Deep Learning; CNN, Convolutional Neural Network; M, Million; WSIs, Whole Slide Images; AI, Artificial Intelligence; AUC, Area Under the Curve; ACC, Accuracy; HCC, hepatocellular carcinoma; SLD, Steatotic Liver Disease; mVI, microvascular Invasion; NRI, Net Reclassification Improvement; IDI, Integrated Discrimination Improvemen; OTA, Ochratoxin A; AFB1, Aflatoxin B1; TCGA, The Cancer Genome Atlas; GEO, Gene Expression Omnibus.

Although AI pathology models have pushed the boundaries of noninvasive diagnostics, they remain broadly constrained by etiological bias. Most existing models were trained on East Asian cohorts with hepatitis B virus (HBV)–driven HCC. However, recent multi-omics studies have highlighted distinct oncogenic mechanisms driven by ochratoxin A (19) and aflatoxin B1 (20), as well as novel ferroptosis-related pathways (21). Consequently, models trained exclusively on HBV-HCC are highly likely to fail in accurately capturing the unique stromal remodeling architectures specific to toxin- or MASLD-driven HCC. To resolve this cross-etiological domain adaptation challenge, cutting-edge algorithms are pivoting toward unsupervised domain adaptation (UDA) and transfer learning. These techniques enable the transfer of knowledge weights from HBV models to MASLD-HCC recognition utilizing a minimal number of target samples. Moreover, integrating causal machine learning to disentangle etiology-specific confounders and extract core, pan-etiology pathological features is a critical pathway to achieving cross-etiology model generalization.

## 4 Survival predictive analytics in medical artificial intelligence

Accurate prediction of survival trajectories following therapeutic intervention is fundamental for optimizing follow-up strategies. Systematic meta-analyses have confirmed that machine learning (ML) models outperform traditional methods when leveraging high-dimensional data to predict recurrence after hepatectomy (22). By providing nonlinear risk stratification of individual overall survival (OS) probabilities, ML models yield quantitative evidence to guide clinical decision-making between surgical resection and liver transplantation (23). Furthermore, models such as random survival forests (RSF) (24), ML algorithms customized for large Barcelona Clinic Liver Cancer (BCLC) stage A/B tumors (25), and biochemical pipelines incorporating preoperative plasma ceramide profiling (26) have further optimized disease-free survival (DFS) predictions.

In the domains of locoregional and systemic therapy, automated NLP models have extracted electronic health record (EHR) data to stratify risk following transarterial chemoembolization (TACE) (27–29). Some studies have employed tools like SHapley Additive exPlanations (SHAP) for *post hoc* feature attribution (30) and have integrated biomarkers (e.g., heat shock protein 90 alpha [HSP90α]) into TACE algorithms (31). For advanced HCC, multimodal deep learning models have successfully predicted responses to combined radiotherapy (32), immune checkpoint inhibitor (ICI) monotherapy (33), and ICI-based conversion therapy (34, 35) (**Table 3**).

**Table 3:** Machine learning for survival and treatment prediction in HCC.

Reference	Year	Algorithms	Sample size	Aim	Best results
Xiang et al. (22)	2025	ML/DL	n=11,850	AI performance: PHR prediction	AUC = 0.91
Kim et al. (23)	2025	ML	n=17,090	ML optimizes LT/SR selection	AUC=0.821 (LT) AUC = 0.778 (SR)
Rhaiem et al. (24)	2026	ML	n=722	Predict DFS after HCC resection	AUC = 0.863
Yang et al. (25)	2025	ML	n=1526	Interpretable ML: Large HCC OS	5-year AUC = 0.75
Lei et al. (26)	2025	ML	n= 257	Ceramide-ML predicts HCC recurrence	AUC = 0.85
Keshavarz et al. (27)	2025	ML/DL	n=4,486	AI predicts TACE response	AUC = 0.88
Kiani et al. (28)	2026	DL	27 studies	AI predicts TACE efficacy	AUC = 0.81
An et al. (29)	2025	ML	n=4462	AutoML predicts TACE prognosis	AUC = 0.84
Hao et al. (30)	2026	ML	n=675	Predict early recurrence after TACE	AUC = 0.741
Su et al. (31)	2026	ML	n=2555	Predict TACE benefit, uHCC survival	3-year AUC = 0.804
Xia et al. (32)	2025	Transformer	n=875	TRIM-uHCC: Prognosis, treatment optimization	C-indices = 0.79
Vithayathil et al. (33)	2025	ML/DL	n=310	Radiomics: Atezo/Bev survival	AUC = 0.81
Lin et al. (34)	2025	ML/DL	n=203	Predict ICIs-conversion HCC DCB	AUC = 0.88
Scheiner et al. (35)	2025	ML/CNN	n=241	ML-TILs predicts immunotherapy outcomes	AUC = 0.78
Li et al. (36)	2026	DL	n=576	LLMs vs. Cox: HCC progression	Cox > LLMs (AUC: 0.97 vs 0.858)
Huang et al. (37)	2025	ML/DL	45 studies	Compare ML and Cox regression	ML > Cox (AUC + 0.05)
Cai et al. (38)	2025	ML	n=424	Optimize HCC prognostic predictions	C-index = 0.720

ML, Machine Learning; DL, Deep Learning; CNN, Convolutional Neural Network; AI, Artificial Intelligence; PHR, Post-Hepatectomy Recurrence; LT, Liver Transplantation; SR, Surgical Resection; DFS, Disease-Free Survival; HCC, Hepatocellular Carcinoma; OS, Overall Survival; AUC, Area Under the Curve; C- index, Concordance Index; TACE, Transarterial Chemoembolization; TRIM-uHCC, transformer-based risk-stratification integrated multimodal model for unresectable Hepatocellular Carcinoma; Atezo/Bev, Atezolizumab plus Bevacizumab; ICIs, Immune Checkpoint Inhibitors; DCB, Durable Clinical Benefit; ML-TILs, Machine Learning Tumor-Infiltrating Lymphocytes; LLMs, Large Language Models; Cox, Cox Proportional Hazards Model.

Despite these advances, recent research has provided crucial methodological introspection. A comparative study by Li et al. demonstrated that traditional Cox proportional hazards (CPH) regression models remain more robust and accurate than advanced LLMs in predicting long-term progression in intermediate-to-advanced HCC (36). Subsequent meta-analyses further confirmed that when processing low-dimensional, tabular clinical data (e.g., routine blood counts and liver function tests), complex ML models offer only marginal utility over standard CPH models (37, 38). These findings objectively highlight the necessity of context-aware AI application rather than universal”AI over-engineering” in clinical oncology research. Researchers must align algorithmic complexity with data dimensionality; while computationally expensive neural networks are indispensable for imaging and spatial multi-omics, their use in scenarios dominated by low-dimensional clinical variables may offer limited added value compared to highly interpretable traditional statistical methods.

## 5 Decoding high-dimensional tumor heterogeneity: synergies between spatial transcriptomics and AI

Intratumoral heterogeneity (ITH) in HCC is a core driver of therapeutic resistance and immune evasion. While traditional single-cell RNA sequencing (e.g., Seurat-based dimensionality reduction and clustering) has unraveled cellular diversity, the necessary tissue dissociation obliterates the spatial topological structure of the tumor microenvironment (TME). Conversely, integrating AI algorithms—such as spatial deconvolution, nonnegative matrix factorization (NMF), and graph neural networks (GNNs)—with spatial transcriptomics (ST) enables the precise mapping of spatial cellular communication networks. The feasibility of this approach is underscored by the successful translation of advanced dimensionality reduction and spatial clustering paradigms previously established in other solid tumors, notably lung cancer (39).

Recent pivotal studies have leveraged AI to decode the interactions between malignant hepatocytes and the TME (**Table 4**). For instance, Liu et al. (40) integrated multi-omics data and utilized algorithms like NMF to infer the spatial polarization trajectories of tumor-associated macrophages (TAMs). These AI algorithms captured intricate microenvironmental interaction patterns that traditional multiplex immunohistochemistry (mIHC) struggles to quantify. Specifically, they revealed that HCC clones do not merely adapt passively; rather, they act as active, core drivers within specific spatial niches, actively reshaping local metabolic networks and suppressing antigen-presentation pathways to drive adjacent TAMs toward an immunosuppressive, mixed M1/M2 phenotype (40). Deep spatial sequencing has further delineated the starkly contrasting transcriptomic architectures between the tumor margin and the core (41, 42).

**Table 4:** AI and spatial transcriptomics in HCC.

Reference	Year	Algorithms	Sample size	Aim	Best results
Bica et al. (39)	2026	ML	19 pts (72,475 cells)	Assess lung cancer heterogeneity	AUC = 0.966 ACC = 0.761
Liu et al. (40)	2025	ML	n=454	Analyze ITH shaping HCC TME	Hypoxic/PLVAP+ EC/VEGFA+ CAF colocalize; border rich in DC1/CXCL10+/SPP1+ Mac
Li et al. (41)	2025	ML	8 pts (19 ST samples)	Analyze HCC tumor nest heterogeneity	TN and LFN exhibit spatial zonation of metabolic core/immune periphery
Yu et al. (42)	2025	ML	1 pt (6,320 spots)	Analyze spatial HCC immune responses	HCC cells exhibit intense immune responses but significant immune escape
Wang et al. (43)	2025	DL	2,794 cells (GSE166635)	Multitargeted drugs: HCC resistance.	CDK1, CCNB1, TOP2A targets. Sorafenib-CDK1 energy -9.2 kcal/mol
Huang et al. (44)	2025	ML	n=1109	S100A10: Diagnostic, immunotherapy target.	AUC=0.963
Lu et al. (45)	2025	ML	n=988	Identify HCC senescence markers	AUC = 0.956 (AURKA) AUC = 0.952 (BIRC5) AUC = 0.849 (CellAge genes)

ML, Machine Learning; DL, Deep Learning; pts, patients; ST, Spatial Transcriptomics; GSE, Gene Expression Omnibus; ITH, Intratumor Heterogeneity; HCC, Hepatocellular Carcinoma; TME, Tumor Microenvironment; AUC, Area Under the Curve; ACC, Accuracy; PLVAP, Plasmalemma vesicle associated protein; EC, Endothelial Cell; VEGFA, Vascular Endothelial Growth Factor A; CAF, Cancer-Associated Fibroblas; DC1, Type 1 conventional Dendritic Cell; CXCL10, C-X-C motif chemokine ligand 10; SPP1, Secreted phosphoprotein 1; Mac., Macrophage; TN, Tumor Nodule; LFN, Liver Fibrotic Nodule; CDK1, Cyclin Dependent Kinase; CCNB1, Cyclin B1; TOP2A, DNA Topoisomerase II Alpha; kcal/mol, Kilocalories per mole; S100A10, S100 calcium binding protein A10; AURKA, Aurora Kinase A; BIRC5, Baculoviral IAP repeat-containing 5.

In drug discovery, Wang et al. (43) and Huang et al. (44) utilized single-cell transcriptomics combined with AI models to anchor core stemness-related genes (e.g., S100A10) driving tyrosine kinase inhibitor (TKI) resistance within complex cellular communication networks. They subsequently employed molecular docking algorithms to screen for potential multi-target therapeutic agents. Concurrently, ML has successfully elucidated the regulatory networks governing cellular senescence within the HCC immune microenvironment (45).

Although AI-driven spatial multi-omics provides profound mechanistic insights, the exorbitant sequencing costs present a formidable barrier to routine clinical translation. Future translational breakthroughs will depend on utilizing deep learning algorithms to map high-dimensional spatial biomarkers defined by ST back onto universally accessible, routine H&E slides. Specifically, cutting-edge research is exploring AI virtual staining technologies—driven by generative adversarial networks (GANs) or diffusion models—to predict spatial transcriptomic expression distributions directly from H&E images. Simultaneously, researchers are using spatial imputation algorithms (e.g., Tangram or SpaGE) to integrate single-cell sequencing data into foundational multiplex immunofluorescence (mIF) images. Ultimately, establishing this cross-modal inference pipeline is essential for transitioning mechanistic discoveries into practical clinical utilities.

## 6 Emerging applications and technological paradigms of AI in HCC

The macroscopic integration of multimodal and multi-omics data is becoming increasingly systematized (**Table 5**). At the foundational architectural level, the introduction of Transformer-based cross-modal attention mechanisms has empowered frontier AI frameworks to surpass traditional early fusion (simple feature concatenation). These frameworks now achieve highly efficient heterogeneous alignment among CT spatial features, H&E pathological slides, and genomic sequences. This deep semantic interaction mechanism significantly advances the construction of personalized therapeutic networks, such as lactylation modification–driven HCC models (46–48). Regarding data representativeness, ML algorithms have begun identifying customized metabolomic biomarkers in African populations (e.g., an Egyptian cohort), thereby promoting equity in oncological research (49). In drug discovery, deep learning frameworks (e.g., the DLCP pipeline) have streamlined the computational workflow from prognostic biomarker extraction to targeted ligand recommendation (50). Furthermore, “intraoperative AI” is emerging, utilizing CV to dynamically guide surgical incisions and evaluate margins, aiming to lock in optimal intervention strategies during the initial phase of surgery (51). Finally, evaluating the potential of LLMs to provide BCLC-aligned treatment recommendations across national registry cohorts underscores the promising future of AI as a robust clinical decision support tool (52).

**Table 5:** Multimodal integration and multi-omics AI applications in HCC.

Reference	Year	Algorithms	Sample size	Aim	Best results
Jin et al. (46)	2025	ML	n=1,215	Identify HCC subtypes for personalization	AUC = 0.94
Wang et al. (47)	2025	ML	TCGA, ICGC, GEO, CPTAC	AI-multi-omics for HCC personalization	AUC = 0.90
Yang et al. (48)	2025	ML	n=932	Lactylation-regeneration: Identifying core genes	AUC = 0.835
Varghese et al. (49)	2026	ML	n=571	Identify Egyptian HCC biomarkers	AUC = 0.98
Wang et al. (50)	2025	ML/DL	n=1,364	dentify biomarkers, anti-HCC ligands	AUC = 0.903
Chan et al. (51)	2025	ML/DL	n=600	AI: Preoperative planning, outcome prediction	AUC = 0.834
Yang et al. (52)	2026	LLM	n=3,024	Evaluate LLMs for HCC treatment	LLM-physician concordance improves early HCC survival.

DL, Deep Learning; LLM, Large Language Model; ML, Machine Learning; TCGA, The Cancer Genome Atlas; ICGC, International Cancer Genome Consortium; GEO, Gene Expression Omnibus; CPTAC, Clinical Proteomic Tumor Analysis Consortium; HCC, Hepatocellular Carcinoma; AUC, Area Under the Curve; AI, Artificial Intelligence; LLMs, Large Language Models.

## 7 Conclusion

Recent literature from 2025 to 2026 has firmly established AI as a core driver in the methodological evolution of precision oncology for HCC. By implementing VFMs to mitigate imaging domain shifts and deploying spatial deconvolution algorithms to decode metabolic interactions within the TME, AI has advanced beyond superficial pattern recognition to deep, mechanistic feature extraction. Nevertheless, objective evidence demonstrating that traditional statistical models continue to outperform complex LLMs when processing low-dimensional tabular data underscores the necessity for judicious algorithmic selection in clinical practice.

## 8 Future perspectives

Looking ahead, medical AI must move beyond an overreliance on “*post hoc* explainability” tools, such as SHAP or LIME, recognizing that their reliability fluctuates dynamically with data complexity and application scenarios. Although these *post hoc* attribution methods excel at highlighting correlative patterns, they face significant limitations in establishing true causality, are generally not engineered to establish biological causality, and may exhibit pronounced mathematical instability within highly complex nonlinear networks. Future AI applications in hepatology must comprehensively pivot toward “inherently interpretable models”. Recognizing that the optimal AI architecture depends dynamically on the specific clinical scenario—where complex “black-box” neural networks are efficient for raw high-dimensional data extraction but lack clinical actionability—innovations like concept bottleneck models (CBMs) offer a balanced paradigm. For instance, in practice, a CBM applied to HCC digital pathology would not simply output a raw “survival probability” directly from an H&E slide. Instead, the model first predicts intermediate, human-understandable clinical concepts (e.g., the degree of steatosis, presence of microvascular invasion, or specific lymphocyte infiltration patterns). The final prognostic decision is then mathematically derived solely from these transparent concepts. This architecture allows oncologists to verify, or even manually correct, the intermediate clinical variables before making a treatment decision, directly anchoring algorithmic outputs to authentic biological causal mechanisms.

Ultimately, clinical translation cannot remain confined to in silico validation. Recognizing that algorithmic utility fundamentally shifts based on data complexity—where highly engineered AI excels specifically in parsing multidimensional biological networks— these models equipped with multimodal fusion capabilities must be rigorously integrated into prospective “window-of-opportunity” clinical trials or randomized controlled trials (RCTs). In a real-world clinical workflow, such as a Multidisciplinary Team (MDT) tumor board, an AI system could act as a dynamic decision-support node. For instance, by instantly synthesizing a patient’s spatial transcriptomic profile with serial CT scans, the AI could flag sub-visual high-risk recurrence features, prompting the MDT to adjust a standard hepatectomy plan to include adjuvant targeted therapy. Concurrently, as regulatory bodies—such as the US Food and Drug Administration (FDA)—confront nonstatic, “black-box” VFMs that continuously evolve without manual fine-tuning, establishing dynamic evaluation frameworks for Software as a Medical Device (SaMD) remains a core translational barrier that interdisciplinary teams must collaboratively overcome.
